# Semantic Segmentation of Sika Deer Antler Image by U-Net Based on Two-Dimensional Discrete Wavelet Transform Fusion and Multi-Attention Mechanism

**DOI:** 10.3390/ani15101388

**Published:** 2025-05-11

**Authors:** Haotian Gong, Jinfan Wei, Yu Sun, Zhipeng Li, He Gong, Juanjuan Fan

**Affiliations:** 1 College of Information Technology, Jilin Agricultural University, Changchun 130118, China; 2 Jilin Province Intelligent Environmental Engineering Research Center, Changchun 130118, China; 3 College of Animal Science and Technology, Jilin Agricultural University, Changchun 130118, China

**Keywords:** sika deer antler, semantic segmentation, U-Net, 2D-DWT, EMCA

## Abstract

At present, the monitoring technology of the growth status of sika deer antlers faces many challenges in a complex breeding environment (such as light change, object occlusion, etc.). More importantly, an effective method for the segmentation of sika deer antlers is still lacking, which hinders the development of subsequent quality classification of sika deer antlers. In order to fill the research gap and lay a foundation for future sika deer antler quality classification, this paper proposed an improved semantic segmentation model based on U-Net, named SDAS-Net. In order to improve the segmentation accuracy and generalization ability of the model in a complex environment, we introduced a two-dimensional discrete wavelet transform module (2D-DWT) in the encoder head to reduce noise interference and enhance the ability to capture features. In order to compensate for the loss of feature information caused by 2D-DWT, we embedded the Star Blocks module in the encoder. In addition, the efficient mixed channel attention (EMCA) module was introduced to adaptively enhance key feature channels in the decoder, and the dual cross-attention mechanism (DCA) module was used to fuse high-dimensional features in skip connections. To verify the validity of the model, we constructed a 1055-image sika deer antler dataset (SDR). The experimental results show that compared with the baseline model, the performance of the SDAS-Net model is significantly improved, reaching 92.12% in MIoU and 93.63% in the PA index, and the number of parameters is only increased by 6.9%. The results show that the SDAS-Net model can effectively deal with the task of sika deer antler segmentation in a complex breeding environment while maintaining high precision.

## Simple Summary

Monitoring the antler growth status of sika deer is of great significance for sika deer antler grading and sika deer class identification, and it also has a role in promoting the process of intelligent breeding of sika deer. In this study, a new network model for segmentation of sika deer antlers was developed based on the U-Net by incorporating innovative modules and attention mechanisms. The method was evaluated using datasets of antler images from adult sika deer. It not only has high accuracy in segmentation tasks but is also very friendly to hardware resources. This provides the data and technological support for sika deer antler quality assessment and grading.

## 1. Introduction

As a precious medicinal material in the treasure house of traditional Chinese medicine, the appearance of plum deer antler, including primary antler, two-pronged antler, three-pronged antler, and even deformed antler, directly determines the distribution proportion of different products, such as wax, powder, gauze, and bone antler [1]. The distribution of different antler pieces greatly affects the medicinal value, nutritional composition, and final market acceptance and economic value of the sika deer antler [2]. Therefore, accurate and efficient monitoring of the growth status of sika deer antlers is very important to reduce industrial economic losses and improve the efficiency of antler resource utilization. However, for a long time, the quality monitoring of sika deer antlers has mainly relied on artificial sensory evaluation. This traditional method has obvious disadvantages: low efficiency, strong subjectivity, and being easily affected by human experience and fatigue [3]. For example, even experienced technicians can only test a limited number of sika deer antlers per day, and the evaluation criteria vary between different inspectors, resulting in a lack of consistency and objectivity in the results. In addition, manual testing is time-consuming and laborious, significantly increasing labor costs. Therefore, it is urgent to develop an efficient, accurate, and objective monitoring method for the appearance of sika deer antlers, so as to improve the quality classification level of sika deer antlers, identify and eliminate deformed antlers as soon as possible, reduce labor costs, and ultimately promote the standardization and intelligent development of the sika deer industry, so as to provide consumers with higher quality antler products.

Although the morphological evaluation of sika deer antlers is of strategic importance to the industrial economy and efficient use of resources, the existing research still faces significant technical bottlenecks. The artificial dependence and subjectivity of traditional sensory detection methods have made it difficult to meet the urgent need for accurate grading of sika deer in large-scale breeding scenarios. In recent years, semantic segmentation technology has shown great potential in the field of precision agriculture and intelligent recognition, providing a new way to solve the above problems [4]. For example, Liu et al. [5] proposed a novel pig counting network based on improved DeepLabV3+, LA-DeepLabV3, which optimized feature fusion by introducing lightweight attention mechanism and the recursive cascade method, effectively reduced parameter redundancy, and improved segmentation accuracy. Tao et al. [6] proposed a multi-scene bovine target segmentation method based on improved DeepLabV3+, Imp-DeepLabV3+, which showed excellent segmentation performance by replacing the backbone network with MobileNetV2 and combining the Squeeze-and-Excitation Networks module to enhance feature interaction. In addition, Xu et al. [7] proposed an intelligent body weight prediction method for dairy cows based on semantic segmentation and backpropagation neural network. ResNet-101-D combined with SE attention mechanism semantic segmentation model was used to extract body parameters of dairy cows, and BP neural network was used to predict body weight, providing technical support for the development of precision agriculture.

In the research field related to sika deer, Gong et al. [8] proposed a facial recognition model of sika deer based on a visual converter combined with the Dense Net module to extract low-level features, fused local and global features through pixel multiplication, and finally recognized them through a pretrained Vision Transformer structure, achieving 97.68% recognition accuracy. In addition, Gong et al. [9] also proposed a sika deer facial recognition model based on SE-Res Net, which further improved the recognition performance by reducing network depth and introducing the SE module to optimize feature extraction. However, the technical focus of the above studies was mainly on individual identification, and the morphological characteristics of antlers have not been explored in depth [10]. In view of the particularity of antler morphological assessment, the existing methods still face three major challenges. First, the surface texture of antlers is complex, and the flume interference is serious, and the traditional feature extraction methods are susceptible to noise. Second, the morphological gradient and continuity exist between deformed antlers and normal antlers, which lead to the blurring of segmentation and classification boundary. Finally, considering the actual needs of mobile farm deployment, it is necessary to find an optimal balance between model accuracy and computational resource consumption.

In order to effectively deal with the above challenges and meet the urgent need for intelligent segmentation and detection of sika deer antlers in sika deer farms, this paper innovatively proposes a new semantic segmentation model named SDAS-Net. Under the premise of ensuring high segmentation accuracy, the SDAS-Net model focuses on optimizing the overall structure of the network, significantly reducing the number of model parameters and computational complexity, so that it can be more efficiently deployed in resource-limited mobile devices. The main contributions of this paper are summarized as follows:

- 2D-DWT module: It innovatively constructs two-dimensional discrete wavelet transform module to generate feature enhancement maps with more discriminating power through multi-scale feature extraction, dense join, residual join, and feature splicing operations, effectively suppress image noise, and significantly improve the model’s ability to capture and characterize key features.
- Star Blocks module: In view of the problem where discrete wavelet transform may lead to slight information loss, the Star Blocks module is introduced to effectively improve the model’s accurate segmentation performance for different sizes and shapes of sika deer antlers by enhancing the feature extraction ability.
- EMCA module: The introduction of the EMCA module significantly enhances the model’s adaptive adjustment ability to feature channel weights, improves the semantic expression ability of features, and helps the model focus on key information areas in the image more effectively.
- DCA module: Aiming at the potential semantic gap between the encoder and the decoder, as well as the detailed information that may be ignored in the encoder feature extraction process, the DCA module is innovatively designed to further improve the overall performance of the deer antler segmentation model by improving the efficiency and effect of feature fusion without significantly increasing the computational burden.
- High-quality antler dataset: A high-quality dataset containing diverse real farming environments and deer antlers of adult sika deer was constructed for model training and performance evaluation, providing valuable data support for in-depth research in related fields.

## 2. Materials and Methods

### 2.1. Materials

#### 2.1.1. Data Sample Collection

**Figure 1:**
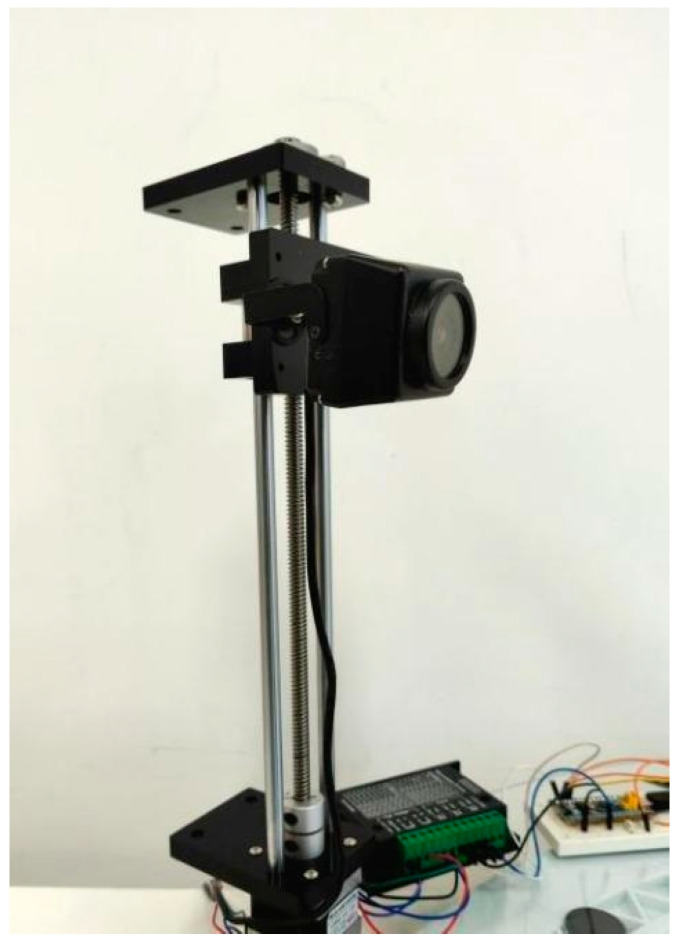
High-resolution 1080p camera.

The experimental data for this study came from the breeding base of plum deer in Shuangyang District, Changchun City, Jilin Province, China. The base, known for its high-quality sika deer population and standardized breeding management, provided a high-quality and representative sample of antler images for this study. Image acquisition was carried out in a natural breeding environment to simulate a real deer antler monitoring scenario. As shown in Figure 1, we used a high-resolution 1080p HD camera to balance image detail and data processing efficiency, close to the level of monitoring equipment that may be applied in actual farms.

**Figure 2:**
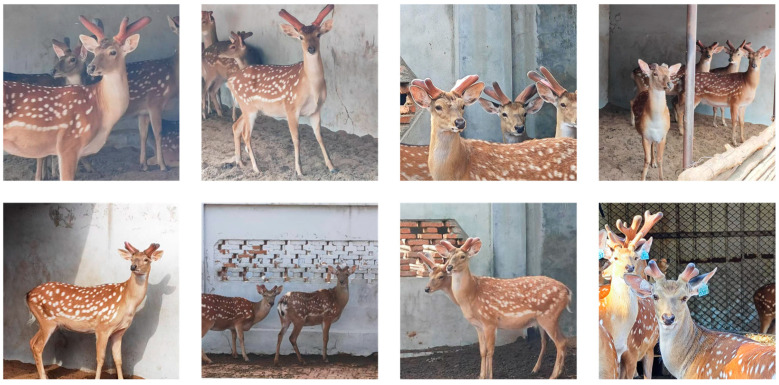
Part of the SDR dataset. We paid special attention to the following scenes when we collected the images: (1) Shooting angle when objects were partially occluded or overlapped; (2) Shooting from the side under sufficient lighting conditions; (3) Shooting in a bright-light environment; (4) Shooting in a low-light environment.

The images are saved in 1920 × 1080 high-resolution JPG format and captured from multiple angles, including the angles of the front and side. The multi-angle shooting strategy aims to comprehensively capture the visual characteristics of different growth stages and different forms of antlers, so as to enhance the diversity of the dataset and the generalization ability of the model. In order to ensure the accuracy and efficiency of antler area labeling, we selected the graphic labeling software Labelme version 5.2.0. Labelme software is widely used in the field of computer vision because of its advantages of simple operation and universal annotation format. By using Labelme software, the sika deer antler area in each image was manually annotated at the fine pixel level by a professional annotator to generate a binary mask image. Using this method, a total of 1055 deer antler image data were created and divided into an 8:2 ratio to train a validation set containing 844 images and a validation set containing 211 images. In addition, we collected 100 images as a test set for visualization comparison of multiple models and feature visualization comparison with generation of heatmaps. Part of the dataset is shown in Figure 2.

**Figure 3:**
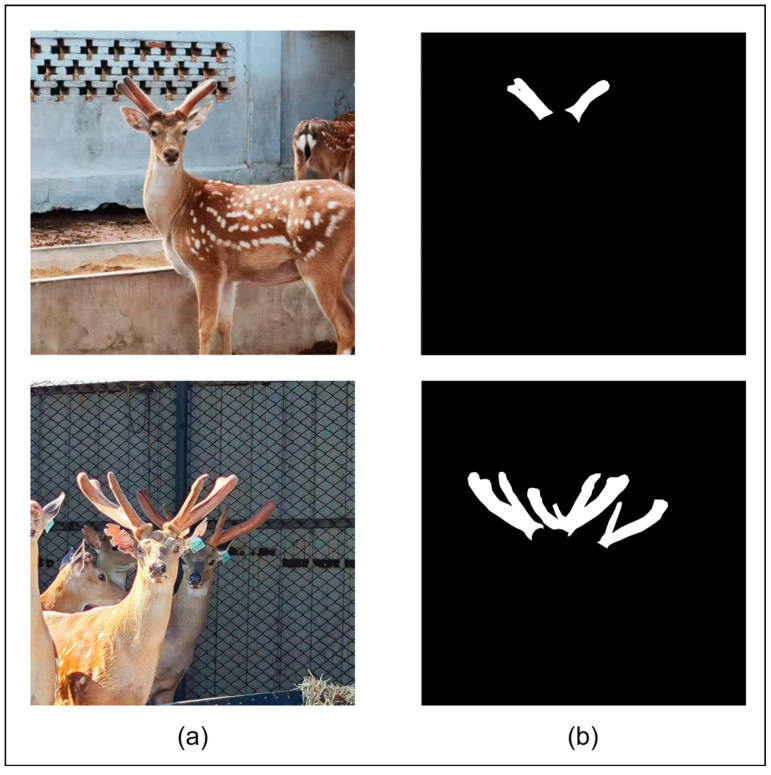
Image labeling: (**a**) Original image; (**b**) Image labeling result (black area as background, white area as sika deer antlers).

In the generated binary mask image (as shown in Figure 3), the black area with a pixel value of 0 represents the image background, and the white area with a pixel value of 255 accurately depicts the outline and range of the sika deer antlers, forming an accurate true label for model training.

#### 2.1.2. Dataset Enhancement

In a real sika deer breeding environment, the image data will inevitably be affected by a variety of adverse factors, such as dynamic changes in natural lighting conditions (including changes in light intensity and angle), complexity of the farm environment (such as image blurring caused by dust, water fog, etc.), and background changes caused by seasonal factors. These factors work together to make the acquired original image data show a high degree of complexity and diversity but may also introduce noise and artifacts, which poses a potential challenge to the generalization ability and robustness of the model. In order to effectively improve the environmental adaptability and anti-interference ability of the model and avoid the phenomenon of over-fitting of the model on the limited dataset, a series of targeted data enhancement techniques were adopted in this study to simulate various degradations and changes in images that may occur in real farming scenes, so as to expand the diversity of the dataset and improve the generalization performance of the model.

Specifically, we applied the following data enhancement strategies to the training set of the dataset:

- Random affine transformation, including rotation, translation, and scaling at random angles. Affine transformation is designed to simulate small changes in camera angle and antler pose to enhance the robustness of the model to changes in the shape and direction of the target.
- Brightness and contrast adjustment: randomly adjusting the brightness and contrast of the image. Brightness and contrast adjustment is designed to simulate the effect of different light intensity and light conditions on image quality, so that the model can adapt to the task of deer antler segmentation in different light environments.
- Compound noise model addition: overlaying multiple types of noise, such as gaussian noise and salt and pepper noise. The introduction of the composite noise model aims to simulate the possible interference factors, such as dust, debris, and sensor noise, in the breeding environment and improve the segmentation performance of the model in the noisy environment.

**Figure 4:**
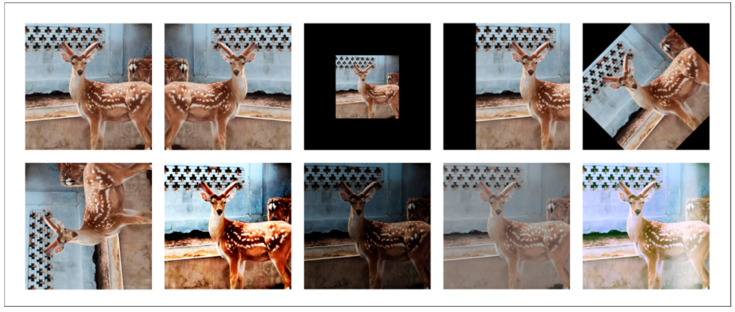
Illustration of partial data enhancement effects in SDR dataset.

Through the comprehensive application of the above data enhancement techniques, the original dataset was effectively expanded. To further harmonize the input image size while taking into account computational efficiency and feature information retention, all images were uniformly adjusted to 512 × 512 pixels. Finally, the size of the SDR dataset after data enhancement was expanded to 3587 images, which significantly improved the diversity of the dataset and the number of training samples of the model and provided a richer data basis for model training. The example results of data enhancement are shown in Figure 4, which visually demonstrates the diversity of the enhanced images. The detailed distribution of the enhanced dataset is summarized in Table 1. It lists in detail the number of images in the original training set, augmented training set, validation set, and test set.

### 2.2. Model Improvement

**Figure 5:**
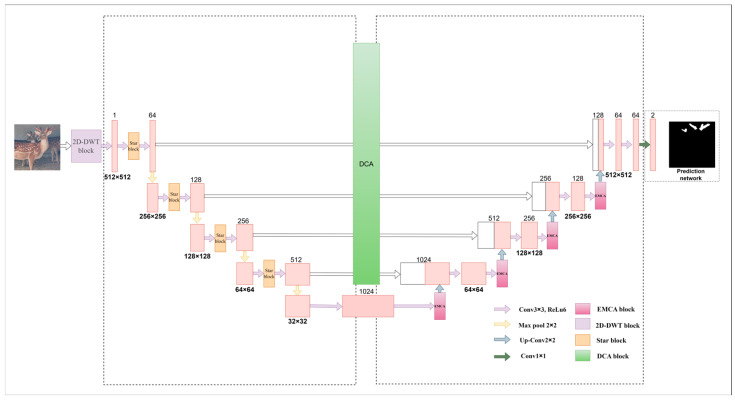
SDAS-Net framework diagram.

The U-Net [11] architecture has achieved remarkable success in the field of image segmentation with its excellent feature extraction capability, effectively alleviating the problem of detail loss common in traditional methods. However, in the face of complex scenarios, especially in environments such as sika deer farms, U-Net models often need deeper and more complex network structures to ensure segmentation performance, which inevitably leads to a significant increase in the number of model parameters and a decrease in training and reasoning speed, which makes it difficult to meet the demand for model efficiency in practical applications. In view of the limitations of the above U-Net model in complex scenarios, we innovatively propose an improved U-Net model named SDAS-Net. The core innovation of the SDAS-Net model lies in its multi-faceted optimization strategy for the limitations of U-Net architecture in the task of deer antler segmentation in complex farming environments. Firstly, the model innovatively introduces a 2D-DWT module at the input end to achieve noise removal and multi-scale feature enhancement, which lays a high-quality data foundation for subsequent feature extraction. Secondly, in the encoder stage, the ultra-lightweight Star Blocks module is embedded to enhance the feature extraction ability at a very low computational cost, compensate for the information loss that may be caused by 2D-DWT, and improve the feature representation ability of the model for different forms of antlers. Then, in the decoder stage, an innovative EMCA attention mechanism is introduced to adaptively retain and enhance the semantic information of key feature channels to improve the segmentation accuracy. Finally, in order to solve the semantic difference in skip connection, a dual cross-attention mechanism (DCA) is introduced to fully integrate multi-scale features between the encoder and the decoder, enhance the efficiency and effect of feature fusion, and improve the ability of the model to capture details of sika deer antlers. Through the synergy of the above series of innovative modules, the SDAS-Net model significantly improves the efficiency and robustness of the model while ensuring high segmentation accuracy. Figure 5 shows the overall improvement approach and framework.

#### 2.2.1. 2D-DWT Module

The two-dimensional discrete wavelet transform is a mathematical tool that decomposes signals or images into components of different frequencies and directions. It is widely applied in fields such as image restoration, adverse weather removal, snow–fog removal, and feature extraction [12,13,14,15]. The two-dimensional discrete wavelet transform can decompose the image into sub-bands of different scales and resolutions, effectively improve the multi-scale and non-linear representation ability of feature extraction, and reduce the noise information [16]. The input image is decomposed into one low-frequency component and three high-frequency components by discrete wavelet transform, as shown in Formula (1):

(1) $$\left\{\begin{array}{c} cA\left(x,y\right)=\sum_{m}\sum_{n}f\left(m,n\right)\cdot \phi \left(\frac{m-2x}{2}\right)\cdot \phi \left(\frac{n-2y}{2}\right)\\ cH\left(x,y\right)=\sum_{m}\sum_{n}f\left(m,n\right)\cdot \phi \left(\frac{m-2x}{2}\right)\cdot \psi \left(\frac{n-2y}{2}\right)\\ cV\left(x,y\right)=\sum_{m}\sum_{n}f\left(m,n\right)\cdot \psi \left(\frac{m-2x}{2}\right)\cdot \phi \left(\frac{n-2y}{2}\right)\\ cD\left(x,y\right)=\sum_{m}\sum_{n}f\left(m,n\right)\cdot \psi \left(\frac{m-2x}{2}\right)\cdot \psi \left(\frac{n-2y}{2}\right) \end{array}\right.$$

**Figure 6:**
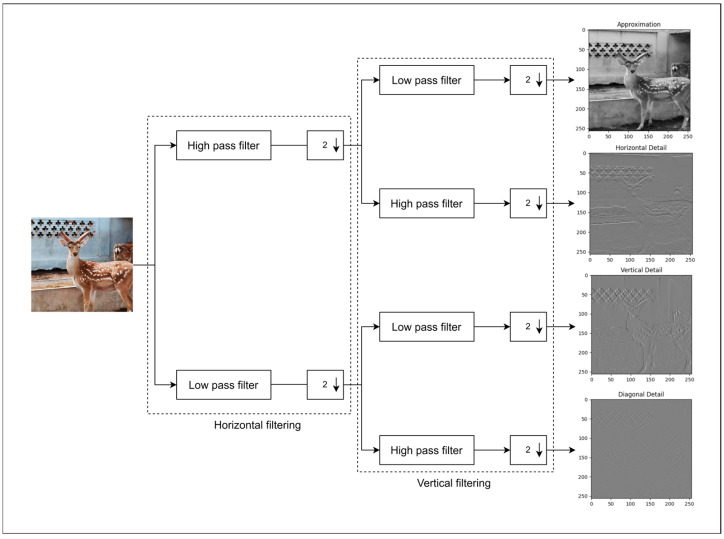
Flow chart of two-dimensional discrete wavelet transforms.

In the formula, cA represents the approximate sub-bands, which can also be expressed as LL, that is, the low-frequency part; cH, cV, cD, respectively, represent the detail sub-bands of the image in the horizontal and vertical diagonal lines and can also be expressed as LH, HL, HH, that is, the high-frequency part. f (m, n) represents the two-dimensional signal value corresponding to the position (m, n). ϕ is the scale function of wavelet transform, which is used to extract the low-frequency information of the image. ψ is a wavelet function used to extract the high-frequency information of the image. The two-dimensional discrete wavelet transform flow of this study is shown in Figure 6 below.

**Figure 7:**
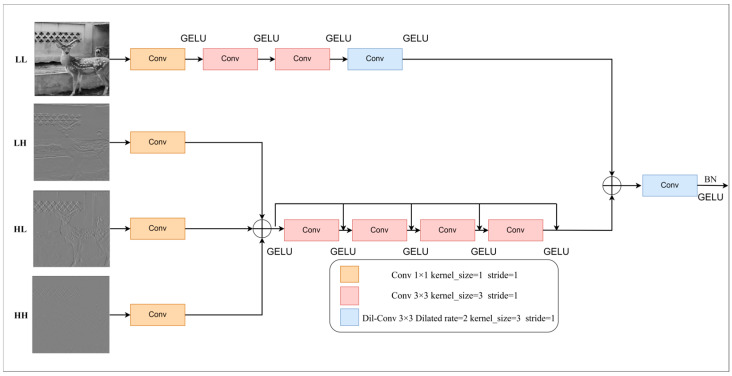
The 2D-DWT module structure diagram.

In this paper, a 2D-DWT module is constructed to extract effective features through multi-scale components, enhance non-linear expression ability, and reduce the impact of data noise on model training. Since the low-frequency information reflects the overall structure of the image, a larger receptive field is needed to capture the global information, while the high-frequency information reflects the details of the image, and a smaller receptive field is needed to focus on local information [17]. In this paper, a variety of convolutions are used to extract low-frequency features, and dilated convolution [18] is used to enlarge the receptive field to enhance the model’s understanding of the global context. For extracting high-frequency features, a multi-convolution architecture based on dense connection and residual connection is designed to enhance local sensing ability by combining low- and high-level features with dense connection. Residual learning focuses on the difference between lower level features and higher level features. In this module, all 3 × 3 convolution kernels have a size of 3 and a step size of 1, while the 1 × 1 convolution kernel size is 1, and its step size is 1. The dilated convolution kernel has a size of 3 and a dilated rate of 2. This structure can capture the global structure and local details of the image at the same time and focus on the key features to achieve the purpose of reducing the number of parameters, improving the reasoning speed, and improving the operation efficiency. The 2D-DWT module structure diagram is shown in Figure 7.

The module adopts a two-branch design: the low-frequency branch extracts the low-frequency features using 1 × 1 convolution, two 3 × 3 convolutions, and 3 × 3 dilated convolution. The high-frequency branch first concatenates the three high-frequency components and then uses 3 × 3 convolution and normalization layer to extract the high-frequency features. Finally, after concatenation of the features of the two branches, down-sampling is performed using 3 × 3 dilated convolution to match the network architecture. The final convolutional layer is followed by BN, and the activation function uses GELU [19] to improve efficiency and stability. The relevant mathematical formula is shown in the following equation:

(2) $$\left\{\begin{array}{c} F_{L}=GELU\left(Dil-Conv\left(GELU\left(Conv2\left(GELU\left(Conv2\left(GELU\left(Conv1\left(I_{LL}\right)\right)\right)\right)\right)\right)\right)\right)\\ F_{H}=X_{1}+GELU(Conv2(X_{1}+GELU(Conv2(X_{1}+GELU(Conv2(X_{1}+\\ GELU(Conv2(Concat(Conv1(I_{LH}),\;Conv1(I_{HL}),\;Conv1(I_{HH}))))))))))\\ F_{O}=GELU(Dil-Conv\left(Concat\left(F_{L},{\begin{array}{c} F \end{array}}_{H}\right)\right)) \end{array}\right.$$

#### 2.2.2. Feature Extraction Branch

In this study, the model is built based on U-Net architecture, and the encoder part adopts 3 × 3 convolution layer and 2 × 2 maximum pooling layer. The Star Blocks module is introduced to eliminate the shortcomings of traditional convolutional networks in high-dimensional non-linear feature representation [20]. Star Blocks achieves high-dimensional feature mapping through star operations, does not increase computational complexity, recursively increases feature dimensions, and obtains richer and more expressive features in low-dimensional inputs, which is crucial for fine feature capture in semantic segmentation [21].

**Figure 8:**
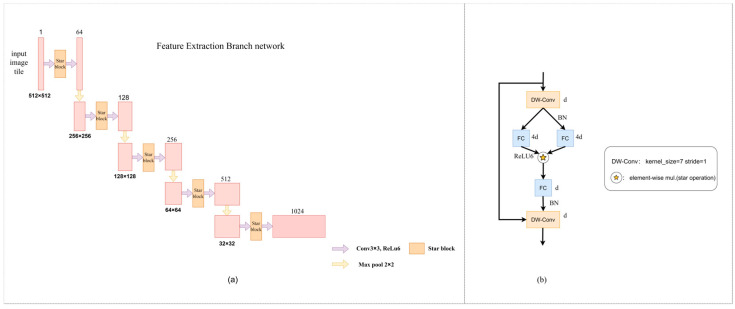
(**a**) Improved feature extraction branch network architecture; (**b**) Structure of Star Blocks.

The improved feature extraction branch network architecture in this paper is shown in Figure 8a, which indicates that the feature extraction branch network architecture consists of a convolution layer and Star Blocks. The role of the architecture is to perform feature extraction (Figure 8b). The Star Blocks module in this study is placed between two 3 × 3 convolutions of the original architecture. In order to improve efficiency, batch normalization is used instead of layer normalization, and it is placed after depth-wise separable convolution [22] to facilitate fusion operations in the inference stage. By adding depth-wise separable convolution at the end of each block, reasonable design of depth and width can significantly improve the feature extraction effect and enhance the semantic segmentation accuracy. The specific parameters are set as follows. The channel expansion factor is 4, and the network width is doubled step by step. The activation function is ReLU6. The common convolution kernel has a size of 3 and a step size of 1. The depth-wise separable convolution kernel has a size of 7 and a step size of 1. The maximum core size and step size of the pool layer are both 2. By stacking multiple layers, the complexity of the hidden dimensions increases exponentially, and high performance and low latency are achieved without complex network structures and high reference numbers. The calculation process of the Star Blocks module is shown in Formula (3).

(3) $$F=Conv\left(ReLU6\left(Conv\left(DW-Conv\left(X\right)\right)\right)⊙ReLU6\left(Conv\left(DW-Conv\left(X\right)\right)\right)\right)$$

In the mathematical formula of the above module, after passing through the module, the feature is F, with DW-Conv representing the depth-wise separable convolution, ⊙ representing the star operation, and X representing the original feature. After the feature X is subjected to the depth-wise separable convolution operation, the result of the linear transformation is then non-linearly transformed via ReLU6 activation function, respectively. Then, the fused features are generated via star operation, and finally, the features F are generated via channel-by-channel convolution operation.

#### 2.2.3. Feature Fusion Branch

In convolutional neural networks (CNNs) [23], the gradual application of convolutional layers will lead to the reduction in image resolution, resulting in the loss of detailed information, and affect the segmentation accuracy of small objects. Therefore, integrating features of different levels in the training stage is helpful to improve the accuracy of multi-scale object detection. The original feature fusion branch enriches feature extraction by synthesizing feature maps of different levels, but it only fuses feature maps via simple up-sampling and layer-by-layer splicing, which lacks the modeling of the relationship between channels and has limited adaptability to complex scenes.

**Figure 9:**
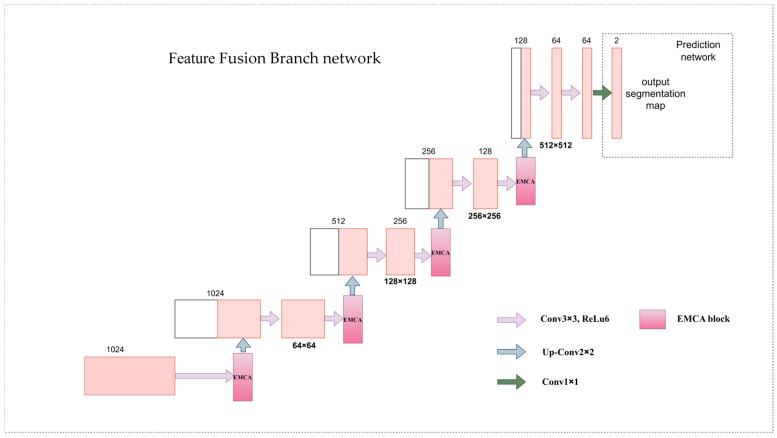
Improved feature fusion branch network architecture.

Therefore, this paper introduces an innovative EMCA module to dynamically adjust channel weights, enhance the importance of modeling feature channels, and improve the multi-scale information processing capability and segmentation performance in complex scenes. The improved feature fusion branch network architecture is shown in Figure 9. After up-sampling, the feature maps are spliced, and then, the updated deep features are generated through convolution operation and then passed through the EMCA module. This process is gradually completed, enabling the prediction network to effectively utilize the main feature maps generated by the U-Net backbone.

#### 2.2.4. EMCA Module

The EMCA module implements the channel attention mechanism in a lightweight manner, which significantly improves the model’s ability to adjust the feature channel weights, enhances the semantic expression of the features, and enables the model to better capture the key information in the image [24]. The specific process is as follows. The module is divided into two branches: the global branch and the local branch. The global branch first performs global average pooling (GAP) on the input feature map and compresses it into a feature vector of channel dimension; then, after reshape operation, the channel attention map is generated via a one-dimensional convolutional layer and then via un-average pooling (UNAP). Local branching first performs local average pooling (LAP) on the input feature map and then generates the channel attention map through one-dimensional convolutional layer after reshape operation. It then implements the reshape operation to adapt it to the subsequent operations. Finally, the global branch features are fused with the local branch features and are restored to the original spatial dimensions through the UNAP operation, which is multiplied by the obtained features and the original features to refine feature representation. A multiplication operation is performed to refine the feature representation. The related operations are shown in Equation (4).

(4) $$F_{1}=F⊙Concat(Conv\left(GlobalAvgPool\left(F\right)\right),Conv\left(LocalAvgPool(F\right)))$$

In the above equation, $F_{1}$ and F are the refined and original input features, respectively; ⊙ is the multiplication operation; and Conv is the one-dimensional convolution.

**Figure 10:**
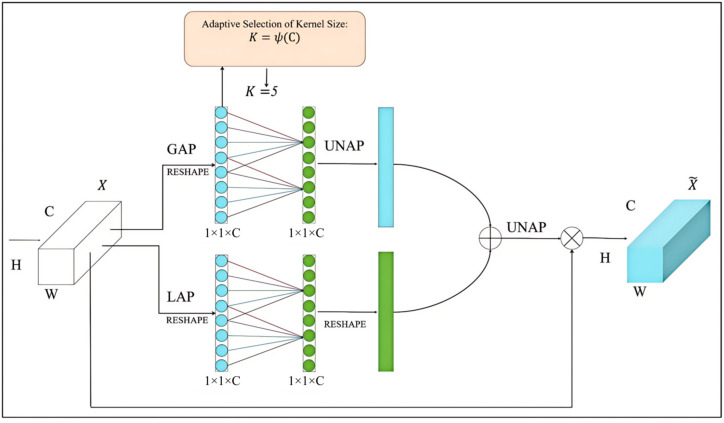
EMCA module architecture.

The core advantage of EMCA modules is efficiency. The module can adaptively enhance the key feature channels in the antler image, keep texture information, reduce the influence of irrelevant channels, optimize the feature representation, and improve precision and reasoning speed. This design is suitable for a resource-limited situation in a real farming environment, and it improves the performance of the model without increasing the number of parameters. Its structure is shown in Figure 10.

#### 2.2.5. DCA Attention Mechanism Module

U-Net passes the features extracted by the encoder to the decoder via the skip connection to recover the details lost during feature extraction. However, as the depth of the network increases, the features extracted by the encoder may ignore detailed information (such as the sika deer outline), resulting in a semantic gap between the encoder and the decoder. To this end, this paper introduces a dual cross-attention module (DCA) in the skip connection, which integrates channel cross-attention and spatial cross-attention to enhance the decoder’s use of shallow features, so as to improve the precision of deer antler segmentation.

**Figure 11:**
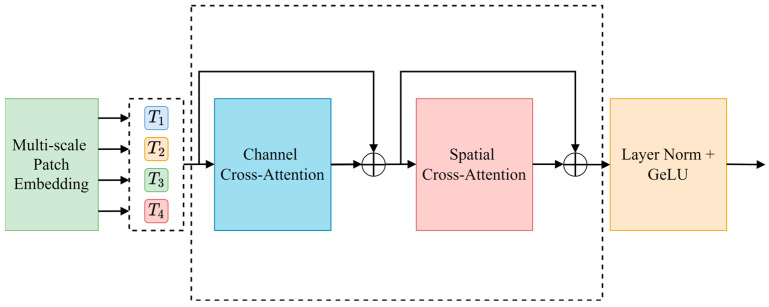
DCA module architecture.

The DCA module receives the multi-scale features output by the encoder and captures the dependency between the features through channel cross-attention and spatial cross-attention mechanisms. After each cross-attention module, the features are fused via a point-by-point summation layer, then processed via batch normalization and GELU activation function, and finally connected to the corresponding decoder. The specific structure of the DCA module is shown in Figure 11.

Since the multi-scale feature extraction module of each coding block has different dimensions of the feature map output, in order to realize the subsequent use of channel and spatial cross-attention mechanisms, two-dimensional average pooling is used to convert the multi-scale feature map into fixed-size tokens, and 1 × 1 depth-wise separable convolution is used to map them on the flattened two-dimensional patch. The detailed explanation is to multiply the 1 × 1 × C convolution kernel point by point with the corresponding C channels of the input feature map and then add the results of these products to produce the final feature representation. This calculation process is described in detail in Formula (5).

(5) $$\left.T_{i}=DWConv{1D}_{F_{i}}\left(Reshape\left(AvgPool{2D}_{F_{i}}\left(F_{i}\right)\right)\right)\right.$$

where $T_{i}$ represents the i-coding sequence (i = 1, 2, 3, 4), which is the feature map of the coding block in the first stage. The reshape operation is to flatten each patch into a one-dimensional vector in a format suitable for depth-wise separable convolution. $AvgPool{2D}_{F_{i}}$ is the average pooling of $F_{i}$. The size and step size of the pooled kernel are $P_{i}^{s}$, where P is the side length of the pooled window, with S indicating the step length. DW-Conv is the depth of 1 × 1 separable convolution, the purpose of which is to project the pooled feature map $F_{i}$.

**Figure 12:**
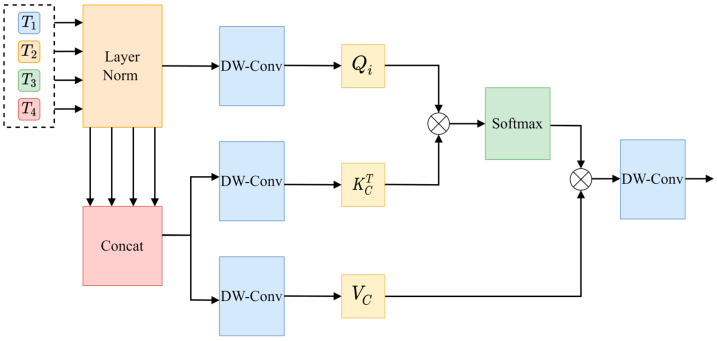
Architecture of channel cross-attention module.

Channel cross-attention focuses on the relationships between channels in the feature graphs of different coding stages and adjusts the weight of each channel by capturing the correlation between these channels. In detail, the more features the weighted channels have, the more important they are considered. On this basis, the features of each channel are weighted to generate the output, so as to generate the final output result. The structure of this process is illustrated in detail in Figure 12.

First, the input layer of the encoding sequence is normalized, and then, $T_{c}$ is obtained via splicing along the channel dimension pairs to $T_{i}$ as Query (Q), and it is used to generate Key (K) and Value (V). $Q_{i},K_{C},V_{C}$ is then generated via linear projection using a depth-wise separable convolution of 1 × 1, as shown in Equation (6).

(6) $$\begin{array}{c} T_{C}=Concat\left(T_{1},T_{2}{,T}_{3}{,T}_{4}\right)\\ Q_{i}=DWConv_{Q_{i}}\left(T_{i}\right)\\ K_{C}=DWConv_{K_{C}}\left(T_{C}\right)\\ V_{C}=DWConv_{V_{C}}\left(T_{C}\right) \end{array}$$

Since channel cross-attention requires channel dimension, $Q_{i}$, $K_{c}$, $V_{C}$ are transposed, so that the channel dimension can be used as the computational dimension of the attention mechanism. The operation of channel cross-attention is shown in Equation (7).

(7) $$CCA\left(Q_{i},K_{C},V_{C}\right)=Softmax\left(\frac{{Q_{i}}^{T}K_{C}}{\sqrt{C_{C}}}\right){V_{C}}^{T}$$

In the above formula, $\frac{1}{\sqrt{C_{c}}}$ is the scaling factor, whose function is to maintain the stability and consistency of the result of the dot product, and it determines the weight of the feature channel through the similarity between $Q_{i}$ and $K_{c}$. Normalization is performed via the softmax function to generate the attention weight values, and finally, the output is projected via depth-wise separable convolution, and four new coding sequences $T_{1}^{c},\;T_{2}^{c}{,T}_{3}^{c}{,T}_{4}^{c}$ are output via a point-by-point summation layer.

**Figure 13:**
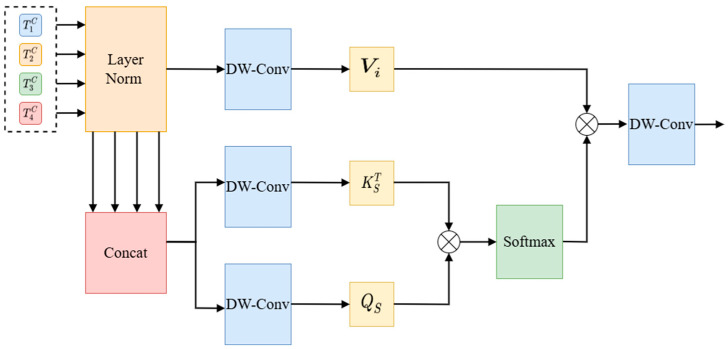
Spatial cross-attention module architecture.

The spatial cross-attention mechanism focuses on capturing the spatial dimension of the input data and enables the model to focus on key areas in the image by calculating the attention weight of each pixel. The spatial cross-attention mechanism is shown in Figure 13.

Different from cross-channel attention, the generated $Q_{S}$ and $K_{S}$ after concatenation $T_{C}^{c}$ are used, and the generated $V_{i}$ after normalization is used, as shown in Equation (8).

(8) $$\left.\begin{array}{c} Q_{S}=DWConv_{Q}\left(T_{C}^{c}\right)\\ K_{S}=DWConv_{K}\left(T_{C}^{c}\right)\\ V_{i}=DWConv_{V}\left(T_{i}^{c}\right) \end{array}\right.$$

In the above formula, DW-Conv is the 1 × 1 depth-wise separable convolution. The formula below shows the spatial cross-attention (Equation (9)).

(9) $$\left.SCA\left(Q_{S},K_{S},V_{i}\right)=Softmax\left(\frac{Q_{S}{K_{S}}^{T}}{\sqrt{d_{k}}}\right)V_{i}\right.$$

$\frac{1}{\sqrt{d_{k}}}$ is the scaling factor. The output of the DCA is obtained by using depth-wise separable convolution. Finally, feature fusion is carried out via the point-by-point summation layer, and the output result is normalized via layer and GELU processing. Finally, the four outputs of the DCA module are connected to their corresponding decoding blocks via up-sampling.

### 2.3. Evaluation Indicators

In order to comprehensively evaluate the performance of the model in the deer antler segmentation task, this paper adopts the evaluation indices commonly used in the field of semantic segmentation: pixel accuracy (PA) and mean intersection over union (mIoU).

Pixel accuracy (PA) is concerned with measuring the accuracy of the model’s classification of each pixel in the semantic segmentation task, that is, the proportion of the number of pixels correctly classified by the model to the total number of pixels. In the deer antler segmentation task, high pixel accuracy means that the model can more accurately distinguish the pixels of the deer antlers from the pixels of the background or other objects, reducing the cases where the pixels of the background or other objects are mistaken for the deer antlers. Its calculation Formula (10) is as follows.

(10) $$PA=\frac{TP+TN}{TP+TN+FP+FN}$$

True positive (TP) refers to a case where the model correctly predicts plum antlers, and the reality also reflects plum antlers, while false positive (FP) refers to a case where the model incorrectly predicts plum antlers, but it is actually some other object. False negative (FN) refers to a case where the model incorrectly predicts some other object, but it is actually plum antlers, and true negative (TN) refers to a case where the model correctly predicts some other object, and the reality also reflects some other object.

mIoU is an index that focuses on the segmentation accuracy of a model in different categories of semantic segmentation tasks. The intersection over union (IoU) value ranges from 0 to 1, with higher values indicating better segmentation performance of the model. The mIoU is the average of the IoU values for all categories. In the task of deer antler segmentation, we usually pay attention to mIoU50 (mIoU when the IoU threshold is 0.5, especially the accuracy of matching the segmented region with the real region) and MIOU50-95 (the average value of mIoU when the IoU threshold is from 0.5 to 0.95, and the step size is 0.05). It indicates performance under different IoU thresholds and more comprehensively reflects the average performance of the model under different IoU thresholds. Its calculation Formula (11) is as follows.

(11) $$mIoU=\frac{1}{C}\sum_{i=1}^{C}{IoU}_{i}$$

## 3. Results

### 3.1. Experimental Environment and Parameter Setting

This experiment is built on PyTorch deep-learning framework and executed in Anaconda environment. Table 2 shows the main experimental equipment environment configuration, while Table 3 shows the main hyperparameter settings.

### 3.2. Experimental Results of the SDAS-Net Model

**Figure 14:**
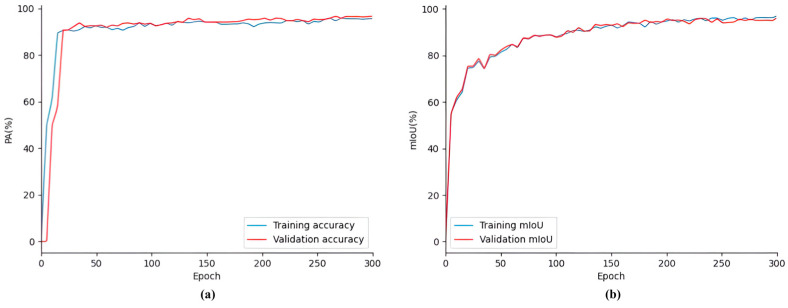
Performance of the SDAS-Net model in the SDR dataset: (**a**) PA curve; (**b**) mIoU curve.

Figure 14 shows the performance of the SDAS-Net model on the SDR dataset. It can be clearly seen in the figure that in the training process, the evaluation index of the model changes with the number of iterations, The PA curves and mIoU curves of the training and validation datasets exhibit an overlapping phenomenon, and there is no significant oscillation. The PA and mIoU values increase rapidly at first; then, the growth rate slows down, and they tend to stabilize. Subsequent training iterations show the convergence of the PA and mIoU value curves. There are no signs of over-fitting, and the outcome of the model training is quite satisfactory. PA and mIoU jointly reflect the overall performance of the model in the detection task. At this stage, the accuracy of the verification set reached 93.63%, and the mIoU was 91.54%.

**Figure 15:**
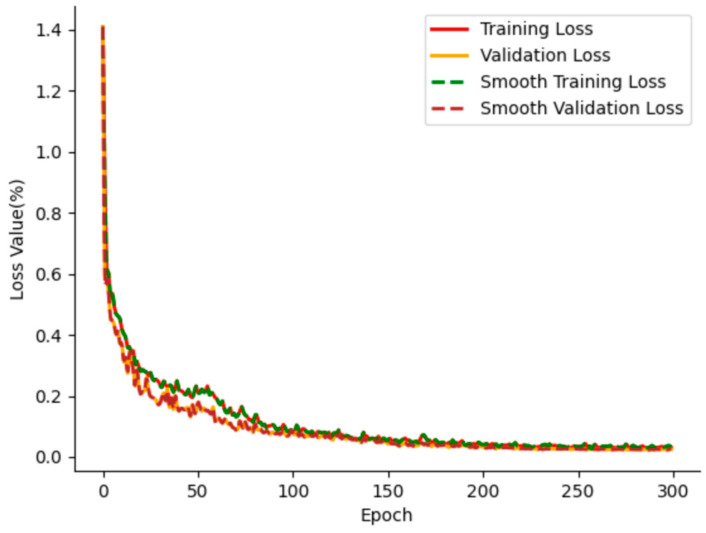
Loss curve of SDAS-Net model.

Figure 15 shows the model during training. The loss curves of the training dataset and the validation dataset overlapped, and there was no excessive oscillation. The loss decreased rapidly at first; then, the rate of decline slowed and then leveled off. After 50 training cycles, the losses stabilized. The subsequent training iterations show the convergence of the loss curve. There is no sign of overfitting, and the model training results are satisfactory. At this stage, the loss value reached 0.05%.

### 3.3. Model Comparison

The PA of SDAS-Net in the SDR dataset reaches 93.63%, which exceeds the PA of U-Net, U-Net++ [23], PsPNet [25], FCN [26], SWFormer [27], and DeepLabv3+ [28] by more than 2.32%, 1.98%, 2.71%, 3.27%, 1.76%, and 1.14%, respectively. Moreover, the mIoU of SDAS-Net reaches 91.54%, which exceeds the mIoU values of U-Net, U-Net++, PsPNet, FCN, SWFormer, and DeepLabv3+ by 6.34%, 6.07%, 6.59%, 7.11%, 4.9%, and 3.04%, respectively. The F1-score of this model reaches 88.9%, which exceeds the F1-scores of U-Net, U-Net++, PsPNet, FCN, SWFormer, and DeepLabv3+ models by 5.73%, 4.92%, 5.96%, 6.51%, 3.59%, and 2.98%, respectively. In terms of model complexity, SDAS-Net has a parameter count of 1.26 × 10^7^, while U-Net, U-Net++, PsPNet, FCN, SWFormer, and DeepLabv3+ have parameter counts of 1.24 × 10^7^, 1.48 × 10^7^, 2.56 × 10^7^, 13.4 × 10^7^, 4.95 × 10^7^, and 4.13 × 10^7^, respectively. In addition, SDAS-Net takes an average of 381.4 ms to complete the segmentation of each image, which is 52.1 ms faster than U-Net. Compared with other models, SDAS-Net has superior performance. Table 4 elaborately presents the performance results of different segmentation models on the validation set. Additionally, to evaluate the true generalization ability of the models, we conducted additional comparative experiments on the test set. Table 5 shows the performance results of different segmentation models on the test set.

### 3.4. Visualization of Segmentation Results

**Figure 16:**
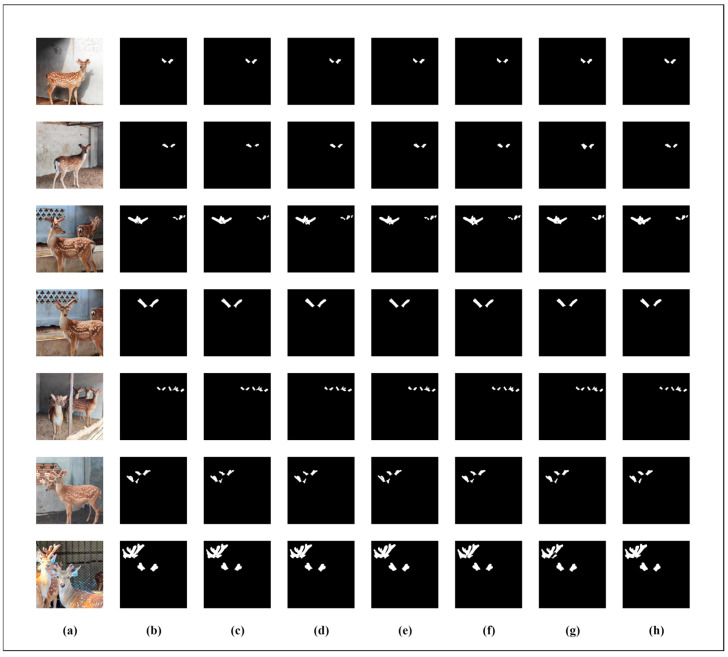
The segmentation results of U-Net, U-Net++, PsPNet, FCN, DeepLabv3+, and SDAS-Net. (**a**) Original image; (**b**) Segmentation results of SDAS-Net; (**c**) Segmentation results of DeepLabv3+; (**d**) Segmentation results of SWFormer; (**e**) Segmentation results of U-Net++; (**f**) Segmentation results of U-Net; (**g**) Segmentation results of PsPNet; (**h**) Segmentation results of FCN.

In order to more intuitively demonstrate the superiority of the SDAS-Net model in the segmentation of Meihua antlers, we visually compared the segmentation results of the U-Net, U-Net++, PsPNet, FCN, SWFormer, and DeepLabv3+ models with the improved SDAS-Net model, respectively, as shown in Figure 16.

The visualization results show that although the DeepLabv3+ model can achieve more accurate segmentation, it is easy to mistakenly segment the deer ear edge into a part of the antlers. When our model predicts images with overlapping antlers, there will be a discontinuity at the overlapping edges of the antlers. This situation is inevitable. However, compared with other models, our model shows significantly better segmentation results when dealing with overlapping antlers and poor lighting conditions. For instance, models such as DeepLabv3+ and SWFormer, when predicting images of overlapping antlers, exhibit obvious phenomena of over-segmentation and under-segmentation, as well as blurred boundaries. The reason is that under the conditions of overlapping antlers and poor lighting, the blurred edges of the antlers are misjudged as the background. In contrast, the SDAS-Net model performs better in the segmentation of antler edges, especially in areas with strong noise and overlap, and its segmentation results are highly consistent with the marked real situation, thus significantly improving the accuracy of antler segmentation.

The results of image segmentation show that the introduction of a two-dimensional discrete wavelet module can improve the extraction of input image features and make the model capable of dealing with the noise information generated by the complex farming environment. In addition, the inclusion of Star Blocks in the encoder and the introduction of EMCA modules in the decoder help the model process the data better, as well as increasing the non-linear representation of the model. The DCA module is added to the skip connection to reduce the feature loss caused by the skip connection and effectively capture the global information, so as to achieve accurate segmentation of sika deer antlers and contours.

### 3.5. Ablation Experiment

To further verify the validity of each module in the SDAS-Net model, we conducted a series of ablation experiments on the SDR dataset. The experiment took the classic U-Net model as the baseline, introduced 2D-DWT modules, Star Blocks, EMCA, and DCA successively, and compared the performance indices of the models under different configurations. The purpose of these ablation experiments was to explore the effects of different modules on the semantic segmentation tasks. Table 6 details the performance of the model with different modules introduced compared to the SDAS-Net model.

**Figure 17:**
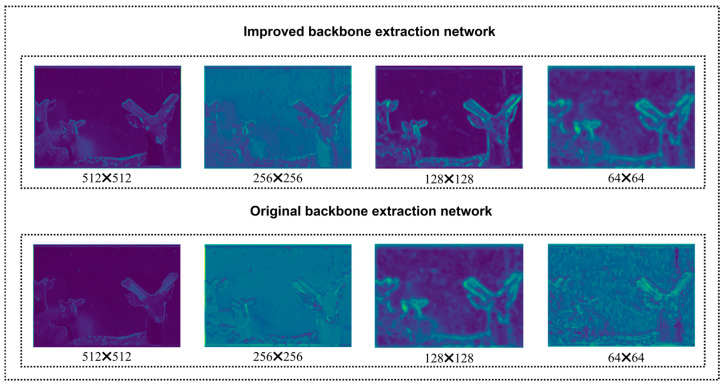
Comparison results of feature map visualization for backbone networks.

Based on the ablation experiment results presented in Table 6, we systematically evaluated the contributions of each proposed module to the model’s performance. On top of the baseline model, when the two-dimensional discrete wavelet transform (2D-DWT) and Star Blocks modules were introduced, the model performance was significantly enhanced. To reveal their mechanisms of action, we visualized the feature maps at the corresponding stages of the encoder, as shown in Figure 17. The feature maps in Figure 17 intuitively demonstrate that after 2D-DWT processing, the high-frequency noise components of the image are effectively suppressed. Meanwhile, the key low-frequency structural information, such as the antler contours, is retained and enhanced. This provides a purer basis for subsequent feature extraction. Subsequently, the Star Blocks module further enhances the feature representation ability. The feature maps output by it exhibit richer texture details and clearer activation of antler boundaries, demonstrating its advantage in capturing multi-scale context information. The combination of these two, verified through feature map visualization, explains the performance gains they bring when dealing with the noisy SDR dataset.

**Figure 18:**
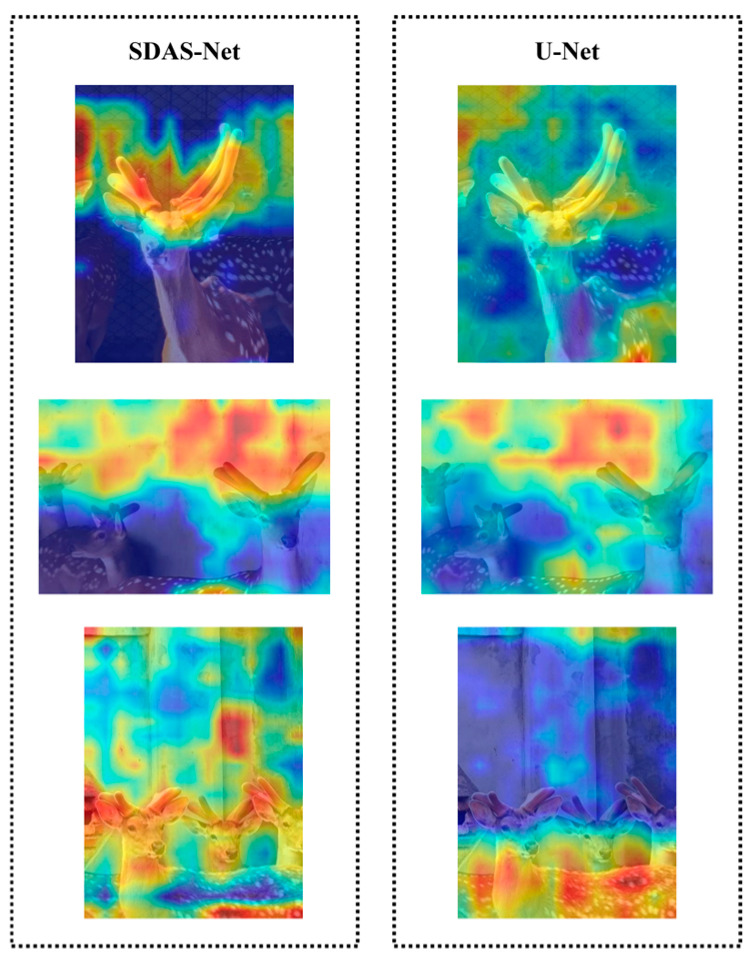
Comparison of thermograms of the improved model with the original model.

Subsequently, we introduced the attention mechanisms EMCA and DCA in the decoder path and skip connections, and the performance metrics were significantly improved once again. To uncover their working mechanisms, we generated the corresponding attention heatmaps, as depicted in Figure 18. Figure 18 shows that after the incorporation of EMCA and DCA, the model can better focus on the feature channels that are more crucial for the segmentation task. The responses corresponding to the antler regions on the heatmap are enhanced. These two modules greatly enhance the model’s ability to distinguish between antlers and the background. Compared with the original model without EMCA and DCA, the attention is significantly concentrated on the antler targets, and the interference from the background region is effectively suppressed. This visually demonstrates that the improved model, through its cross-attention mechanisms, successfully fuses features at different levels in the skip connections and guides the model to precisely focus on key semantic information, thus explaining the performance improvement it brings.

Ultimately, the SDAS-Net model, which integrates all the innovative modules, attains optimal performance. This is attributed to the synergistic effect among its components, as corroborated by the above-mentioned visual analysis. As can be seen in Figure 17, 2D-DWT and Star Blocks provide high-quality initial features. And as depicted in Figure 18, EMCA and DCA utilize these features to achieve a more precise attention focus and information fusion. This complete chain, from denoising at the input end to enhancing feature representation and then to fusing at the decoding end and focusing on key regions, collectively improves the model’s ability to accurately segment sika deer antlers in complex backgrounds.

## 4. Discussion

In this study, an improved U-Net model, SDAS-Net, is successfully proposed to achieve high-precision semantic segmentation of sika deer antlers, and its excellent performance is verified on our self-built SDR dataset. Through detailed ablation experiments, we fully demonstrate the significant improvement in model performance by innovative modules, such as 2D-DWT, Star Blocks, EMCA, and DCA. SDAS-Net can not only accurately and effectively separate various forms of deer antlers, but more importantly, it can effectively distinguish the overlapping areas of deer ears and deer antlers that are easily confused, showing good adaptability to complex farming environments, which lays a solid foundation for practical application.

The SDAS-Net model achieves an excellent balance between pixel-level segmentation accuracy and model parameter number. Compared with the original U-Net, SDAS-Net can maintain and even improve the segmentation accuracy while effectively reducing the number of model parameters, significantly reducing the memory consumption, and improving the inference speed. This advantage is mainly due to the key modules we introduce. First, the 2D-DWT module improves the feature representation capability and robustness of the model from the source through multi-scale feature extraction and efficient noise reduction. Second, the DCA module further enhances the model’s ability to capture and segment the fine contours of antlers through the dual cross-attention mechanism. Notably, although the Star Blocks and EMCA modules may have performance bottlenecks on extremely complex generic datasets, they take full advantage of the enhanced feature extraction and channel selection on deer antler image data preprocessed and optimized by the 2D-DWT module to compensate for potential performance deficiencies. Together, they contribute to improvement in the overall performance of SDAS-Net.

However, there are some limitations in this study. The current SDR dataset is mainly derived from sika deer in specific regions and farms, and although it covers a variety of complex scenarios, the SDAS-Net model may still need to be fine-tuned or further optimized for different species and different growing environments to ensure optimal generalization performance. Although the SDAS-Net model we propose can already handle some mild occlusions and overlaps in the current dataset, effectively distinguishing regions such as antlers and deer ears, we are well aware that in real and more complex breeding environments, antlers may encounter more severe and diverse occlusion challenges. For example, they may be largely covered by the bodies of other deer, fences, or feeding facilities. Effectively dealing with such complex occlusions is the key to improving the model’s robustness and accuracy in practical applications. Therefore, the focus of our future research will be to significantly enhance the model’s ability to handle complex occlusions. First, this involves constructing more challenging occlusion datasets, such as partially erased variants that simulate real-world occlusion scenarios or using generative adversarial networks to synthesize occluders, so as to expand the diversity of training samples. At the model level, we plan to explore integrating or designing modules on the basis of SDAS-Net that can explicitly perceive and handle occlusions. For instance, we can draw on the idea of partial convolution for processing irregular image regions or further optimize the attention mechanism, so that it can infer the occluded parts using the visible context. Through these multi-dimensional and systematic explorations, we hope to build a more robust and accurate antler segmentation model that can cope with complex occlusions in real-world breeding environments. We also plan to extend the application of the SDAS-Net model to more deer species and even other horned animals, such as red deer and reindeer. Meanwhile, we will continue to pay attention to practical application requirements, such as lightweight deployment of the model and real-time requirements, and will further optimize and improve the SDAS-Net model according to these needs, providing more solid technical support for the intelligent upgrading of the antler industry.

## 5. Conclusions

Aiming at the problem of the lack of effective segmentation technology in the existing classification and monitoring methods, this paper innovatively proposed a SDAS-Net model to achieve high-precision semantic segmentation in complex breeding environments. In order to achieve accurate segmentation in complex environments and fill the technical gaps, we designed and integrated 2D-DWT modules in the front end of the model. By means of cavity convolution, multi-scale convolution, dense connection, and residual connection, the module effectively enhanced the feature extraction capability of high- and low-frequency feature maps and exhibited efficient denoising performance. The data preprocessed by the DWT module were more conducive to the advantages of the subsequent Star Blocks and EMCA modules, avoiding the problem of performance degradation in noisy or sparse datasets. To address the problem of feature loss during up-sampling in the original U-Net model, we innovatively proposed the EMCA module, which focuses more on the ability to capture detailed features by combining channel attention at the local and global levels. Aiming at the semantic gap in the original U-Net model, we introduced the DCA module.

This module utilizes the multi-level features of the output of the multi-scale encoder, effectively captures the correlation between channels and spatial pixels through the channel cross-attention and spatial cross-attention mechanisms, adaptively adjusts the feature weights, and thus focuses on the target area of velvet more accurately, significantly improving the segmentation ability of the model on velvet. The experimental results on a self-built SDR dataset containing various forms of antlers and complex backgrounds show that the SDAS-Net model proposed in this paper achieved significant performance improvement. It achieved 93.63%, 91.54%, and 88.9% on PA, mIoU, and F1-Score, respectively, while maintaining a low reference count (1.26 × 10^7^) and fast reasoning speed (381.4 ms). The experimental results fully verify that the SDAS-Net model has the advantages of light weight and high efficiency while maintaining high segmentation accuracy, and it can effectively meet the needs of accurate segmentation of sika deer antlers in complex breeding environments. Especially in the segmentation of complex forms of antlers, such as two-pronged sika deer antlers, the SDAS-Net model shows higher accuracy and faster speed, which fully reflects the huge application potential and economic value of the SDAS-Net model in the sika deer breeding industry and lays a solid technical foundation for the future quality classification and intelligent monitoring of antlers based on accurate segmentation.

## Figures and Tables

**Table 1:** Dataset distribution.

Training Set		Validation Set	TEST SET
Original image	Enhanced image	Original image	Original image
844	3376	211	100

**Table 2:** Experimental environment configuration.

Environment Configuration	Argument
CPU	Intel(R) Xeon(R) Gold 6148 CPU @ 2.40 GHz
GPU	NVIDIA A100
Development environment	PyCharm 2023.2.5
Language	Python 3.8.10
Frame	PyTorch 1.8.10
Operating platform	CUDA 11.7

**Table 3:** Experimental hyperparameter settings.

Experimental Parameter	Argument
Input image size	512 × 512
Epoch	300
Batch	16
Adam learning rate	0.000327
Momentum	0.9
Weight decay	0.0002

**Table 4:** Performance results of different segmentation models on the validation set.

Model	PA (%)	MIoU (%)	F1-Score (%)	Parameters (10^7^)	Time (ms)
U-Net	91.31	85.2	83.17	1.24	433.5
U-Net++	91.65	85.47	83.98	1.48	477.1
PsPNet	90.92	84.95	82.94	2.56	484.6
FCN	90.36	84.43	82.39	13.4	530.7
SWFormer	91.87	86.64	85.31	4.95	272.9
DeepLabv3+	92.49	88.5	85.92	4.13	276.2
Our	93.63	91.54	88.9	1.26	381.4

**Table 5:** Performance results of different segmentation models on the test set.

Model	PA (%)	MIoU (%)	F1-Score (%)	Parameters (10^7^)	Time (ms)
U-Net	87.62	79.61	76.42	1.24	347.8
U-Net++	88.99	81.36	77.59	1.48	406.1
PsPNet	91.88	80.25	84.56	2.56	469.3
FCN	89.98	81.69	87.89	13.4	493.7
SWFormer	91.11	85.85	88.01	4.95	228.9
DeepLabv3+	91.67	89.15	88.82	4.13	213.6
Our	92.96	89.94	89.78	1.26	316.4

**Table 6:** Comparison results between the model performance of different modules and the SDAS-Net model.

Number	U-Net	2D-DWT	Star Blocks	EMCA	DCA	PA (%)	mIoU (%)
1	√	√				92.38	89.92
2	√		√			91.51	86.37
3	√			√		91.92	88.26
4	√				√	92.42	90.1
5	√	√	√	√	√	93.63	91.54

## Data Availability

All new research data are presented in this contribution.
